# Aberrant Frequency Related Change-Detection Activity in Chronic Tinnitus

**DOI:** 10.3389/fnins.2020.543134

**Published:** 2020-10-23

**Authors:** Abdoreza Asadpour, Mehran Jahed, Saeid Mahmoudian

**Affiliations:** ^1^School of Electrical Engineering, Sharif University of Technology, Tehran, Iran; ^2^ENT-Head and Neck Research Center, Hazrat Rasoul Akram Hospital, Iran University of Medical Sciences, Tehran, Iran; ^3^The Five Senses Health Institute, Iran University of Medical Sciences, Tehran, Iran

**Keywords:** tinnitus, electroencephalogram, mismatch negativity, multi-feature paradigm, change detection, echoic memory, sensory-memory hypothesis

## Abstract

Tinnitus is the perception of sound without the occurrence of an acoustic event. The deficit in auditory sensory or echoic memory may be the cause of the perception of tinnitus. This study considered the mismatch negativity (MMN) to investigate the potential difference between and within groups of persons with normal hearing (NH) and tinnitus. Using an auditory multi-feature paradigm to elicit the MMN, this study considered the MMN peak amplitude at two central frequencies for two MMN subcomponents. These central frequencies were 1 and 5 kHz, which the latter was closer to the perceived tinnitus frequency in the group with tinnitus. The deviants were higher frequency, lower frequency, higher intensity, lower intensity, duration, location (left), location (right), and gap. The pure tone audiometry (PTA) test and distortion product otoacoustic emissions (DPOAE) test showed no meaningful difference between the two groups. For the frontal subcomponent, the mean amplitudes of the MMN peak for the two groups illustrated less negative meaningful MMN peak amplitudes in the group of persons with tinnitus. For the supratemporal component at 5 kHz central frequency, amplitudes were lower for the group of persons with tinnitus, whereas for the central frequency of 1 kHz, most deviants exhibited meaningful differences. Additionally, within-group comparisons indicated that mean amplitudes for both groups were more negative at the central frequency of 1 kHz for the frontal MMN subcomponent. In comparison, the supratemporal component illustrated a lower peak amplitude at 5 kHz central frequency in the group of persons with tinnitus and no difference in the NH group, which is a unique observation of this study. Results of the between-groups are in accordance with previous studies and within-group comparisons consider the probability of decreasing the change detection capability of the brain. The results of this study indicate that increasing the frequency of the stimuli close to the tinnitus perceived frequencies decreases the prediction error, including the prediction error of the silence. Such a decrease may cause the prediction error of the spontaneous neural activity in the auditory pathway to exceed the silence prediction error, and as a result, increases the probability of occurrence of tinnitus in higher frequencies according to the predictive coding model.

## Introduction

### Tinnitus and MMN

Tinnitus is the common symptom of “ringing in the ear,” the scientific definition of which is sound perception without an acoustic event (Baguley, 2002). Persons with tinnitus generally report differing sensations, ranging from soft pitch to high frequencies. This symptom induces sleeplessness and a drastic lack of concentration. The symptoms affect more than 45 million people in the United States, and approximately 20% of the world population (Tinnitus Archive, 2017). Ample evidence suggests that the mechanism of tinnitus involves maladaptive plasticity in both classic and non-classic auditory pathway (Eggermont, 2003; Noreña and Eggermont, 2003). The non-classical auditory pathways are multi-modal sensory inputs to the auditory and limbic systems, and extralemniscal pathways (Saunders, 2007). Other studies suggest a deficiency in the paralimbic system, limbic system, the engagement of attention and memory, or the output of cochlea may play a role in the generation of tinnitus (Rauschecker et al., 2010; Husain et al., 2011; Schaette and McAlpine, 2011; Kraus and Canlon, 2012). These deficiencies imply the involvement of distress, emotion, attention, memory, and other networks in the tinnitus occurrence. Among the theories describing this symptom, are deficiencies in auditory sensory-memory and pre-attentive central auditory processing mechanisms (Mahmoudian et al., 2013).

Mismatch negativity (MMN) is an auditory event-related potential (ERP) component that includes the presence of a deviant-tone stimulus among frequent standard-tone stimuli. The brain generates an early negative response waveform approximately 140–210 ms after a stimulus violates a regularity in the preceding auditory tones. The way the deviant tone differs from the standard tone characterizes different domains of the MMN. As such, the deviant tone must differ in at least one physical feature compared to the standard tones (Davalos et al., 2005).

There are two main interpretations of the MMN mechanism. In the *sensory-memory hypothesis*, MMN is an index of auditory sensory or echoic memory, context-dependent information processing primarily at the level of auditory cortices, and sound discrimination accuracy (Umbricht and Krljes, 2005; Kujala et al., 2007; Niznikiewicz et al., 2009). According to this hypothesis, standard stimuli create a neural representation and a memory trace. The change detection system elicits the MMN by finding a different stimulus (Näätänen and Alho, 1995; Näätänen et al., 2005). Using the sensory-memory hypothesis, Friston (2005) introduced a hierarchal predictive error model and suggested that standard inputs may cause a prediction of lower input in higher levels of the brain model. Accordingly, this prediction transfers to lower levels through backward connections and when the prediction is incompatible with the input of lower levels, a prediction error occurs which elicits the MMN (Friston, 2005). Many previous studies have used the sensory-memory hypothesis to interpret the results of such investigations (Näätänen et al., 2005; May and Tiitinen, 2010; Fishman, 2014). Another interpretation of the MMN mechanism is the *adaptation hypothesis*, where a regular stimulus causes an attenuated response in a specific neural population, sensitive only to that stimulus. A non-regular (deviant) stimulus causes a distinct neural population to fire, which, due to less frequent stimulation, is not subject to attenuation. The neuronal population produces a stronger response and subtraction of these responses can thus elicit the MMN (May et al., 1999; May and Tiitinen, 2010).

Previous studies suggest at least two subcomponents for the MMN. Nose-referenced mastoid electrodes can detect the supratemporal component (Näätänen et al., 1978, 2007; Giard et al., 1990; Fishman, 2014). The supratemporal component may reflect the sensory memory change detection aspect of the MMN (Giard et al., 1990; Tse et al., 2013), whereas the frontal component associates with an involuntary attention-switching process (Giard et al., 1990; Fishman, 2014). The electroencephalogram (EEG) signal detects the frontal component in the right hemisphere of the brain, although some studies point to the involvement of the left inferior frontal gyrus (IFG) (Giard et al., 1990; Fishman, 2014; Hofmann-Shen et al., 2020).

### Literature Review

In 1996, Jacobson and colleagues compared the selective auditory attention between the individuals with and without tinnitus symptoms. The study included 37 participants with tinnitus who had normal hearing (NH) sensitivity to frequencies under 1500 Hz and 15 NH participants. All participants experienced four stimuli conditions with 500 and 1000 Hz frequencies. The MMN latency difference between groups was non-significant, while the group with tinnitus showed significantly larger MMN amplitude. The authors observed this effect *a posteriori* and suggested that there is more attentional processing of the stimuli in the group with tinnitus (Jacobson et al., 1996). To test the Lesion-Edge (LE) hypothesis, Weisz et al. conducted a study involving 15 participants with tinnitus and high-frequency hearing loss, alongside a group of 15 persons with NH. The standard stimulation represented two conditions: (1) an LE condition in which the frequency of the stimulation was the lowest frequency before the hearing threshold decreased and (2) a control condition in which the frequency of the stimuli was one octave below the LE frequency. The study utilized deviants of 1, 2, and 4% lower frequencies than the standard stimulation. Results showed that in the group with tinnitus, as the distress was intensified, the difference between mismatch-related sources became more negative. The study reported significantly higher normalized amplitude of MMN in the LE condition for the 1% deviants and lower normalized amplitude for 2% deviants in the group with tinnitus compared to the NH group, while the results showed no significant difference in the control condition (Weisz et al., 2004). A later study utilized the MMN to verify habituation deficits in 25 NH participants with severe tinnitus compared to 13 participants with NH as the control group. The standard stimulus was a single tone sound with a frequency of 1000 Hz and an intensity of 70 dB sound pressure level (SPL), and the frequency of the deviant stimulus was 1100 Hz. The study compared the mean latency and amplitude of the MMN between two groups, where mean latencies in both ears were significantly higher for the control group (Holdefer et al., 2013). A different study investigated the effects of the acoustic stimulus on the auditory sensory memory. The study involved 28 participants with chronic tinnitus and 33 persons in a healthy control group. A multi-feature paradigm for five deviants inclusive of frequency, intensity, duration, location, and silent gap extracted the MMN component and the amplitude, latency, and area under the curve of the MMN component. Results showed significantly more negative mean amplitude and higher area under the curve of the MMN component in frequency, duration, and silent gap deviants for the control group compared to the group of persons with tinnitus. In contrast, other parameters and deviants did not exhibit significant differences between groups. The results indicated that the silent gap MMN was most affected by tinnitus and also a possible deficit in auditory memory mechanisms involved in pre-attentive change detection in participants with tinnitus (Mahmoudian et al., 2013). To investigate the effect of transcranial magnetic stimulation (rTMS) on persons with subjective tinnitus, before and after treatment, Yang and colleagues extracted MMN and compared groups of persons with and without tinnitus while examining the treatment effect on that MMN. Twenty tinnitus patients with NH or mild hearing loss and 16 NH control group took part in the experiment. The investigation used a regular pure tone 1000 Hz stimulus for 85% of the trials and a target pure tone of 1500 Hz stimulus in 15% of the trials to elicit the MMN. The amplitude of the MMN was significantly lower in the group with tinnitus before the treatment compared to the control group and also lower compared to the group with tinnitus after rTMS treatment, evaluated through the Fz channel. The study concluded that tinnitus is due to the impairment of an initial sensory memory mechanism in the temporal lobe (Yang et al., 2013). The attentional bias in individuals with decompensated tinnitus was the focus of another study by Li and colleagues. The study involved seven participants with unilateral chronic subjective tinnitus and hearing loss in the tinnitus-affected ear and 14 individuals with NH as the control group. The standard stimulation was a pure tone 8500 Hz frequency sound, and the study used two pure tone deviants with 8000 and 9000 Hz frequencies to elicit the MMN. The study reported that the 9000 Hz frequency induced a significant MMN in the group with tinnitus, and the amplitude of ERP between 90 and 150 ms was significantly higher in the group with tinnitus compared to the control group. The study proposed that persons with tinnitus may utilize automatic processing of acoustic stimuli and therefore may allocate more cognitive resources for such processing (Li et al., 2016).

### Problem Specification and Assumptions

As explained in Section “Tinnitus and MMN,” maladaptive plasticity in the primary and non-primary auditory pathway may result in tinnitus. Hence, investigating and searching for a parameter that can extract the maladaptive plasticity will be of importance. Previous studies suggest that the MMN is an index of central auditory system plasticity (Näätänen, 2008; Garrido et al., 2009), and studying the changes of the MMN in tinnitus patients may provide essential information about the underlying mechanisms of tinnitus.

Many types of stimulus changes can elicit the MMN, including pitch, frequency, duration, and intensity. Subtracting the ERP waveform caused by a standard event from the ERP waveform of the deviant event usually produces the MMN. The difference wave reflects the processing of change between the two stimulus events and depicts a negativity presented over frontotemporal electrode locations that peaks around 100–200 ms post-stimulus presentation. More accurately, exact parameters depend on the nature of the deviance, the task paradigm, the stimuli, and the population under study (Näätänen, 1995).

### Analytical and Statistical Models and Methods

Mismatch negativity is a component of the auditory ERP. Thus, investigation of the MMN may utilize extant analytical approaches employed for the study of ERP components.

#### Signal Averaging

The amplitude of the ERP components is usually smaller than the amplitude of the background ongoing EEG (Mouraux and Iannetti, 2008). Therefore, it is essential to enhance the signal to elicit the desired ERP. Dividing the signal into epochs and averaging these epochs time-locked to a specific latency is a proposed method to improve the ERP components (Burns et al., 2013). The method requires repeating the event enough times and assumes that the ERPs are *stationary* (i.e., the mean value of the ERPs is constant over epochs). Therefore, the averaging procedure does not affect the ERP components. Since background ongoing EEG has zero-mean noise and is uncorrelated to the ERPs, averaging cancels out most of the background EEG noise (Mouraux and Iannetti, 2008). The averaging scheme is,

$$\begin{array}{c} y_{1}=ERP_{1}+{\text{η}}_{1}\\ y_{2}=ERP_{2}+{\text{η}}_{2}\\ .\\ .\\ .\\ y_{\text{n}}=ERP_{\text{n}}+{\text{η}}_{\text{n}} \end{array}$$

where *y* is the signal acquired for each epoch and η is background ongoing EEG, and therefore the averaged signal is,

$$y_{a\,v\,e\,r\,a\,g\,e\,d}=E\,R\,P_{a\,v\,e\,r\,a\,g\,e\,d}$$

Previous studies generally utilized two common methods of averaging. The stimulus-locked averaging method locks to the onset of the stimulus and investigates the sensory and cognitive aspects of the brain. The response-locked averaging scheme locks to the motor response in studies that the participants must respond to the stimuli (Berchicci et al., 2016). The current study utilizes stimuli and does not require a response from the participants, and therefore employs the former method of averaging, namely, the stimulus-locked scheme.

#### Baseline Correction

It is common to measure the amplitudes of the ERP components, including the MMN, relative to the mean of the pre-stimulus baseline (Woodman, 2010). Nevertheless, there may be a systematic pre-stimulus neural activity that may not affect the activity after the onset of the stimulus, and using a mean pre-stimulus baseline will copy this activity into the post-stimulus data (Urbach and Kutas, 2006; Maess et al., 2016). Also, high-pass filters may induce artifacts that affect the baseline and peaks (Acunzo et al., 2012; Tanner et al., 2016). Therefore, a more robust baseline selection is necessary in order to reduce the effects of noise and artifacts.

#### Independent Component Analysis

Independent component analysis (ICA) is a higher order statistical technique that attempts to recover linearly independent components of an observed signal source by reducing the statistical dependence of an observed collection of signals. The scheme can effectively remove artifacts, such as eye blink and electrocardiogram (ECG) signal from the EEG data. Marco-Pallarés et al. implemented the combined ICA-low resolution brain electromagnetic tomography (LORETA) to extract the MMN subcomponents. However, the accuracy of the extracted subcomponents was low due to the limited number of the EEG channels and low accuracy of the LORETA in general (Marco-Pallarés et al., 2005). The use of the ICA is highly effective in performing the blind source separation (BSS) of the EEG for several reasons. The data recorded by multiple electrodes are nominally presumed to be a linear mixture of temporally independent sources arising from spatially fixed areas (Zhou and Gotman, 2005). Two consecutive studies compared common techniques of artifact removal and concluded that the ICA-based methods were effective at separating various types of artifacts from the EEG, without requiring to mask cortically generated signals (Jung et al., 2000a,b).

The basic ICA model has *n* observed random variables, *x*_*i*_, which is a linear combination of *n* statistically independent random sources, *s*_*i*_ (Hyvarinen et al., 2001):

x=iasi1+1asi2+2…+asi⁢n  ni=1,2,…,n

The matrix notation usually presents the model as noted below, where $\vec{x}$ represents the vector of observed signals at any given time, *A* is the mixing matrix of all mixing coefficients, and $\vec{s}$ is a vector of independent source signals at any given time. Next, *W* is defined as the inverse of the non-singular *A* matrix and hence $\vec{x}$ and $\vec{s}$ vectors are (Krishnaveni et al., 2006).

$$\vec{x}=A\,\vec{s}$$

$$\vec{s}=W\,\vec{x}$$

Most of the previous studies mainly utilized frequency deviants to compare the MMN characteristics between groups. These studies neither evaluated nor compared the changes of the MMN features in persons with tinnitus as the standard stimulation frequency changed drastically and moved closer to perceived tinnitus frequency. The current study utilizes a multi-feature paradigm in two central frequencies in order to investigate the features of the MMN between participants with subjective chronic tinnitus and the NH control group and within each group between two central frequencies. These features may explain the occurrence of tinnitus symptoms based on the sensory-memory hypothesis. Section “Materials and Methods” explains materials and methods for implementing the experiments inclusive of the selected paradigm, characteristics of the participants, and the data processing scheme. Section “Results” presents the results of the proposed protocols followed by Section “Discussion,” which provides a comprehensive discussion of the results inclusive of the sensory-memory hypothesis of the MMN mechanism.

## Materials and Methods

### Experimental Setup

Frequently, studies utilize the MMN through a classic two-stimulus auditory oddball implementation. The experimental protocol commonly allows for the random presentation of a rare auditory deviant within a train of repetitive standards. Such an approach has several drawbacks. The most notable of these drawbacks is the allotted time requirements for paradigms, which bounds the responses to only two different deviant types. In a more efficient approach, Näätänen et al. (2004) suggested the use of a multi-feature MMN paradigm, where the MMN from up to five different deviants can be recorded in a relatively short period of time (Fisher et al., 2008). The utilized paradigm illustrated increased sensitivity compared to traditional MMN tasks. These results indicate that as different MMN deviant types probe diverse auditory information processing, the use of a multi-feature MMN paradigm will allow greater accuracy in profiling the deficits (Thönnessen et al., 2008).

### Design

The current study considered a multi-feature paradigm (Näätänen et al., 2004). In the multi-feature MMN paradigm, the standard stimuli were tones of 75 ms duration composed of three sinusoidal partials of 500, 1000, and 1500 Hz as low-frequency stimuli and three sinusoidal partials of 4500, 5000, and 5500 Hz as high-frequency stimuli, which are close to the tinnitus perceived frequency. Here, the intensity of 1000 and 1500 Hz partials, and 5000 and 5500 Hz partials were lower than that of the 500 and 4500 Hz by 3 and 6 dB, respectively.

Additionally, the deviant tones differed from the standard tones in frequency, duration, intensity, perceived location of sound origin, or contained a gap in the middle of the tone. All stimuli were presented through two stereo speakers (except for the intensity deviants) at the SPL of 70 dB with equal phase intensity for each channel. Half of the frequency deviants were 10% higher, while the other half were 10% lower than the standard frequency. Also, half of the intensity deviants were 10% higher, while the other half were 10% lower than the standard intensity. The perceived difference between the standard tone and the location deviant was approximately 90°, and the chance in the perceived location of sound origin was obtained by creating a time difference of 800 μs for half of the location deviants to the right channel and half of the deviants to the left channel. The duration deviant was 25 ms, while the gap deviant was created by removing 7 ms (including 1 ms rise and fall) from the middle of the standard stimulus.

This case-control and comparative study was conducted on adults with chronic subjective tinnitus referred to a tertiary educational referral outpatient center. All participants gave written informed consent, and the protocol was approved by the Medical Research Ethics Committee and the Board of clinical research at ENT and Head and Neck Research Center of Iran University of Medical Sciences (ENT-IUM), Tehran, Iran, with reference number IR.IUMS.REC.1393.9011369004. The study was clearly described to the participants, and the protocols of this investigation were in line with the Declaration of Helsinki.

### Participants

This study utilized 28 participants (22 males and six females, Mean ± 95% CI: 42 ± 5 years old) experiencing chronic tinnitus and 11 individuals as NH control group (eight males and three females, Mean ± 95% CI: 29 ± 8 years old). The age difference between groups was large according to Cohen’s criteria (Cohen, 1988) and significant (*F*_1_,_36_ = 9.562; *p* < 0.01; $\eta_{p}^{2}$ = 0.210; 95% CI [−21.12, −4.38]). Adult persons with a history of chronic tinnitus who were previously admitted to the tinnitus clinic of ENT and Head and Neck Research Center in Hazrate Rasoul Hospital, Iran University of Medical Sciences between December 2015 and March 2017, were included in this study. The inclusion criteria were: (1) definite diagnosis of chronic subjective idiopathic tinnitus (>6 months), confirmed by both an audiologist and an otorhinolaryngologist; (2) age 18–55 years old; (3) normal external and middle ear function checked via otoscopy and tympanometry; (4) Behavioral pure tone audiometry (PTA) threshold levels ≤ 20 dB hearing level (HL) in octave frequencies of 250–2000 Hz and not more than 40 dB HL in frequencies of 4000 and 8000 Hz; (5) ability to read, speak, and write in the Persian language; and (6) agreement to take part in the study and complete the follow-up. The exclusion criteria were: (1) history of chronic neurological or auditory diseases; (2) history of exposure to excessive noise, acoustic trauma, ototoxic agents, head trauma, and whiplash injury; (3) use of neurological/psychiatric medications within the past 3 months; (4) pregnancy or breastfeeding; (5) temporomandibular disorders; (6) receiving treatment for tinnitus within the past 3 months; (7) alcohol or drug abuse; (8) head and neck disease or space-occupying lesion; and (9) tinnitus secondary to a systemic disease.

Subjective measures of loudness, annoyance, and awareness are captured in widely used scales in the form of a visual analog rating scale (VAS) (Landgrebe et al., 2012; Theodoroff and Folmer, 2013). Another method for assessing tinnitus distress is through the use of questionnaires. A handful of self-report questionnaires have been validated for use in participants with tinnitus (Landgrebe et al., 2012). Most established questionnaires, namely, the Tinnitus Handicap Inventory (THI), the Tinnitus Handicap Questionnaire (THQ), and the Tinnitus Questionnaire (TQ) (Heller, 2003; Landgrebe et al., 2012; Theodoroff and Folmer, 2013) yield highly inter-correlated scores. This study used THI and TQ as the questionnaires for evaluating tinnitus distress.

### Acquisition Protocols

The protocol of this study has two stages: (1) behavioral and physiological evaluations and (2) EEG acquisition.

#### Behavioral and Physiological Evaluations

##### Psycho-acoustical evaluations

In this study, psycho-acoustical evaluations were performed to characterize the subjective description of tinnitus. The psycho-acoustical evaluations included pitch matching of tinnitus (PMT), loudness matching of tinnitus (LMT), minimal masking level (MML), and acoustical residual inhibition (RI) procedures. The procedures which were implemented through the TinnED^®^ device were conducted as the criteria to qualify individuals for the study. The TinnED^®^ was designed at the ENT-IUM and met the calibration standards for audiometric equipment as recommended by the American Standard Specification for Audiometers, S3.6-2004.

Pitch matching of tinnitus and LMT were evaluated using external tones which were presented to the contralateral ear by a headphone (Sennheiser, HDA 280). The TinnED^®^ device accomplished the required tasks by recording six channels to reconstruct the most troublesome tinnitus (MTT) with a similar frequency and intensity. The device is a computer-based sound synthesizer with dedicated software that is adapted to the standards for tinnitus assessments. The device provides an opportunity to present required tones and noise with variable frequency and intensity, individually or mixed, in order to synthesize tinnitus. Thus, the mentioned device may provide sound characteristics that are most similar to tinnitus.

Pitch and loudness match tests were performed contralateral to the ear experiencing tinnitus. LMT was obtained at each of the test tone frequencies regardless of the pitch of the tinnitus. The sound level was increased in 1 dB steps until the participant reported that the external tone is just equal to the loudness of the tinnitus. Thereby, the loudness of tinnitus was established according to the dB sensation level (dB SL). The test tone was started below the individual’s hearing threshold in the ascending series of intensity levels to minimize loudness changes of tinnitus. For the tinnitus pitch-match test, a two-alternative forced-choice method was administered. Different pairs of pitch sounds were generated at 15 frequencies (from 125 Hz to 12 kHz) to match the loudness of the tinnitus; then, the pitch was decreased or increased. Next, each participant was asked to identify the tone that best matched the pitch of the participant’s tinnitus. The pitch-match test was performed typically in multiples of 1 kHz. Then, an octave confusion test was administered to improve tinnitus frequency verification. Finally, the loudness obtained at PMT was considered as LMT.

The VAS, Persian TQ (P-TQ) (Daneshi et al., 2005), and Persian THI (P-THI) (Mahmoudian et al., 2011) were obtained from participants to assess the severity of tinnitus. The participants with severe chronic tinnitus having scores more than 44% in P-TQ, 39 in P-THI, LMT of more than 6 dB SL, and scores more than 6 out of 10 points in VAS were enrolled in the study.

##### Physiological evaluation

Distortion Product Otoacoustic Emissions (DPOAE) test was performed (PATH MEDICAL GmbH^1^) using nine different frequency combinations of primary tones (0.05, 1, 1.5, 2, 3, 4, 5, 6, and 8 kHz) to assess cochlear function. The intensity levels were L1 = 55 and L2 = 65 dB SPL and ratios of f1/f2 = 1.22 and the evoked responses for 2f1 − f2 were assessed.

#### EEG Acquisition

Participants seated in an acoustically and electrically shielded room during the EEG recording sessions. Participants were instructed to completely relax their muscles while minimizing movements and eye blinking. Instructions also required the participants to attend, during the data acquisition periods, to a silent nature-based documentary video with Persian subtitles. During the data gathering periods, two loudspeakers presented the auditory stimuli at azimuths of ca. +45° and ca. −45° from the meridian.

**FIGURE 1:**
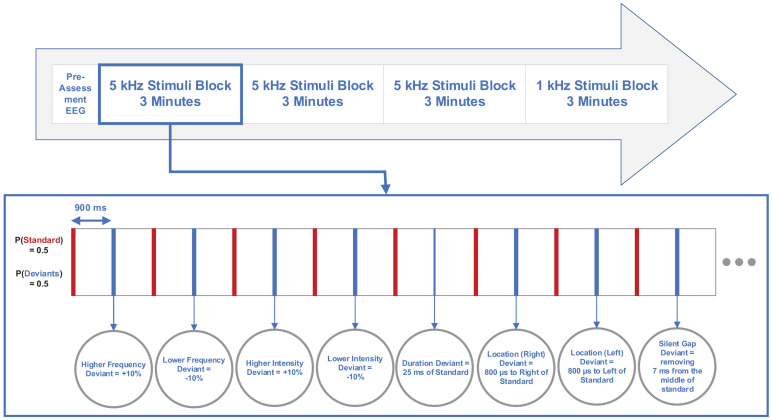
Diagram illustrating multi-feature mismatch negativity (MMN) paradigm and stimuli features.

A total of 200 sounds were presented in each block with four blocks per participant. Three first blocks consisted of 100 deviant tones and 100 standard tones between every two deviants as high-frequency stimuli. The last block contained 100 deviant tones and 100 standard tones between every two deviants as low-frequency stimuli according to the multi-feature paradigm (Näätänen et al., 2004). Additionally, a 3-min preassessment of EEG signal was acquired from each participant under conditions of open eyes, open eyes with hyperventilation, and closed eyes with 1 Hz flashing strobe. The inter-stimulus interval (ISI) was 825 ms. Figure 1 illustrates the multi-feature MMN paradigm and stimuli features.

Electrical brain activities were recorded using 64−channel BRAIN QUICK LTM (Micromed, Italy) referenced to the tip of the nose. Twenty-nine scalp sites (FP1, FPz, FP2, F7, F3, Fz, F4, F8, FT7, FC3, FCz, FC4, FT8, T7, C3, Cz, C4, T8, TP7, CP3, CPz, CP4, TP8, P3, Pz, P4, POz, M1, and M2) were selected according to the International 10−10 system, to place Ag/AgCl electrodes. Two channels (M1, M2) were recorded from individual electrodes placed on the left and right mastoids. A LO1 electrode was placed on the outer canthus of the left eye to provide horizontal electrooculogram (EOG) data and the IO1 electrode on the infraorbital ridge acquired potentials due to blinks and vertical eye movements. Recordings from both EOG electrodes were referenced to the tip of the nose in the unipolar montage. The ground electrode was placed between the Fz and FPz sites. Inter-electrode impedances were kept below 10 kΩ and the sampling frequency was 1 kHz. The online filter was a band-pass filter of 0.4–200 Hz. Neurobehavioral Systems Presentation software (Albany, CA, United States) was used to present the auditory stimuli, and each stimulus onset was automatically recorded by inputting markers within the presentation file and through inserting a pulse trigger in one of the EEG channels.

### Pre- and Post-processing and Analysis

Electroencephalogram data were imported to MATLAB 2013a, and a 3380th-order zero-phase finite impulse response (FIR) filter was used for band-pass filtering on the continuous unsegmented data, with cut-off frequencies (6 dB point) of 0.5 and 20.5 Hz and a transition band of 1 Hz. The ICA with 29 components was applied on the filtered signal to remove eye blinks, ECG and EOG, using the runica infomax algorithm. The FIR filter and ICA were created by the EEGLAB toolbox for MATLAB (Delorme and Makeig, 2004).

After ICA, signals were separated into epochs (950 ms) inclusive of a 50 ms pre-stimulus interval relative to time pulses. The epochs whose channel voltage exceeded ±50 μV were removed. For each participant, standard and deviant evoked responses were averaged, and then the standard average was subtracted from each deviant average in order to determine the MMN wave. The MMN amplitude was the peak negative amplitude occurring during the period of 110 and 200 ms and was labeled under the supervision of a neuroscientist.

#### Baseline Selection

**FIGURE 2:**
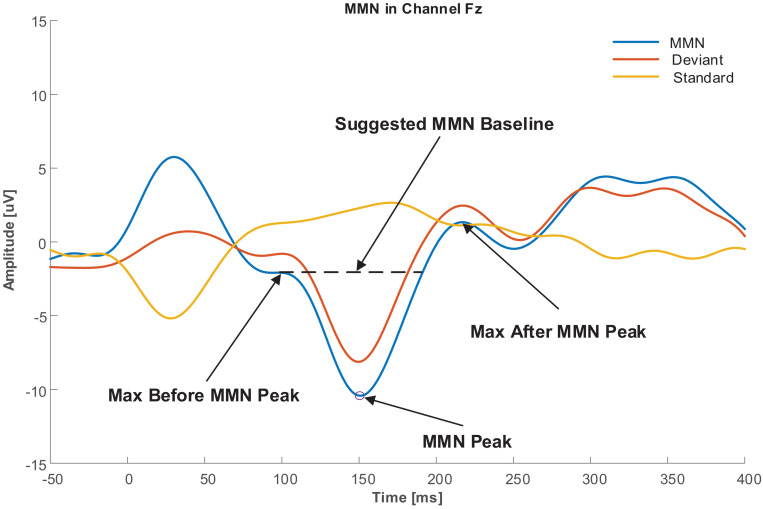
Baseline selection for MMN peak.

As explained in Section “Baseline Correction,” a robust baseline selection that is not affected by pre-stimulus neural activities and other baseline variations is crucial. To achieve this goal, a baseline was proposed in this study with respect to the labeled MMN peak. The baseline was defined as the amplitude of one of the two extremums next to the MMN peak, which had a lower amplitude difference than the MMN peak. Figure 2 illustrates the baseline selection for the MMN peak.

#### Temporal Features

After baseline selection, the amplitude of labeled MMN peaks was calculated for six channels (F3, Fz, F4, FC3, FCz, and FC4) where MMN had the highest activity in the region of interest (ROI) channels extracting the frontal subcomponent of the MMN (Giard et al., 1990; Li et al., 2016). Also, for extracting the supratemporal subcomponent of the MMN, peaks were calculated for two channels, namely, M1 and M2 (Näätänen et al., 1978).

#### Analytical Methods

Five different independent variables were considered for the frontal subcomponents, namely, Groups, Central Frequencies of the stimuli, Deviant Types, Frontality of the EEG channels, and Laterality of the EEG channels. The MMN peak amplitude was the dependent variable. Therefore, a 2 (Group: Tinnitus, NH) × 2 (Deviant central frequency: 1 kHz, 5 kHz) × 8 (Deviant type: Higher Frequency, Lower Frequency, Higher Intensity, Lower Intensity, Duration, Location (Left), Location (Right), Gap) × 2 (Frontality: F, FC) × 3 Laterality (3, z, 4) mixed analysis of variance (ANOVA) was used to investigate the main effects and their interactions in the frontal subcomponent for each temporal feature. Also, a 2 (Group: Tinnitus, NH) × 2 (Deviant central frequency: 1 kHz, 5 kHz) × 8 (Deviant type: Higher Frequency, Lower Frequency, Higher Intensity, Lower Intensity, Duration, Location (Left), Location (Right), Gap) × 2 Laterality (1, 2) mixed ANOVA was used to investigate the main effects and their interactions in the supratemporal subcomponent for each temporal feature since this subcomponent does not include Frontality of the EEG channels. Mixed ANOVA with a critical α of 0.05 was used to evaluate the main effects and interactions. Since the age difference was significant between the groups, the age of the participants was added as a covariate to investigate any age-related effects on the results. The ANOVA was performed using SPSS v. 20 with type IV sum of squares.

Because of noise and artifact contaminations, the database had missing values for some participants. Therefore, in order to utilize the data of all the participants for mixed ANOVA, this study implemented an imputation on the data and replaced the missing values with imputed values (Jakobsen et al., 2017; Kanemura et al., 2018). The method of imputation was linear regression with singularity tolerance of 10^–12^ using five imputations with 1000 iterations. The default sphericity correction method for repeated-measures variables in this study was Huynh–Feldt correction, where ε was higher than 0.75. Otherwise, the Greenhouse–Geisser method was used to correct the inferential statistics of main effects and interactions. Also, the effect size of the comparisons is reported as small, medium, and large according to Cohen’s criteria (Cohen, 1988).

For further investigation, the results of the univariate ANOVA were calculated between the group with tinnitus and the NH control group for 2 (Group: Tinnitus, NH) × 8 (Deviant type: Higher Frequency, Lower Frequency, Higher Intensity, Lower Intensity, Duration, Location (Left), Location (Right), Gap) and within each group for 2 (Deviant central frequency: 1 kHz, 5 kHz) × 8 (Deviant type: Higher Frequency, Lower Frequency, Higher Intensity, Lower Intensity, Duration, Location (Left), Location (Right), Gap). Furthermore, when the Levene’s test of homogeneity of variance was violated, the Kruskal–Wallis H test was implemented to investigate the significant differences between and within groups.

## Results

This study compares the behavioral and physiological results as well as the “Peak Amplitude” features, extracted from the data in the frontal and supratemporal subcomponents. As an overview, behavioral and physiological results are depicted in Section “Behavioral and Physiological Results,” including PTA and DPOAE comparisons. Result of the comparisons between the group with tinnitus and the NH group depicts a small and non-significant difference. Section “MMN Results” presents the inferential statistics of the main effects and interactions of the mixed ANOVA for the independent variables affecting MMN peak amplitude. For between-groups comparisons, the age difference between groups did not significantly affect the results. Furthermore, Section “Between Groups Comparisons” shows that the means of peak amplitudes were less negative for the frontal subcomponent and less positive for the supratemporal subcomponent. Large differences were observed in almost all of the deviants at both central frequencies of 1 and 5 kHz for the group with tinnitus. For within-group comparisons, Section “Within Group Comparisons” indicates that the means of peak amplitudes were less negative for the frontal subcomponent. Furthermore, the means of peak amplitudes were less positive for the supratemporal subcomponent with small to medium differences at the central frequency of 5 kHz compared to deviants at the central frequency of 1 kHz in the group with tinnitus. In the NH group, the means of peak amplitudes for deviants at the central frequency of 5 kHz were significantly less negative, with small differences only for the frontal subcomponent.

### Behavioral and Physiological Results

**FIGURE 3:**
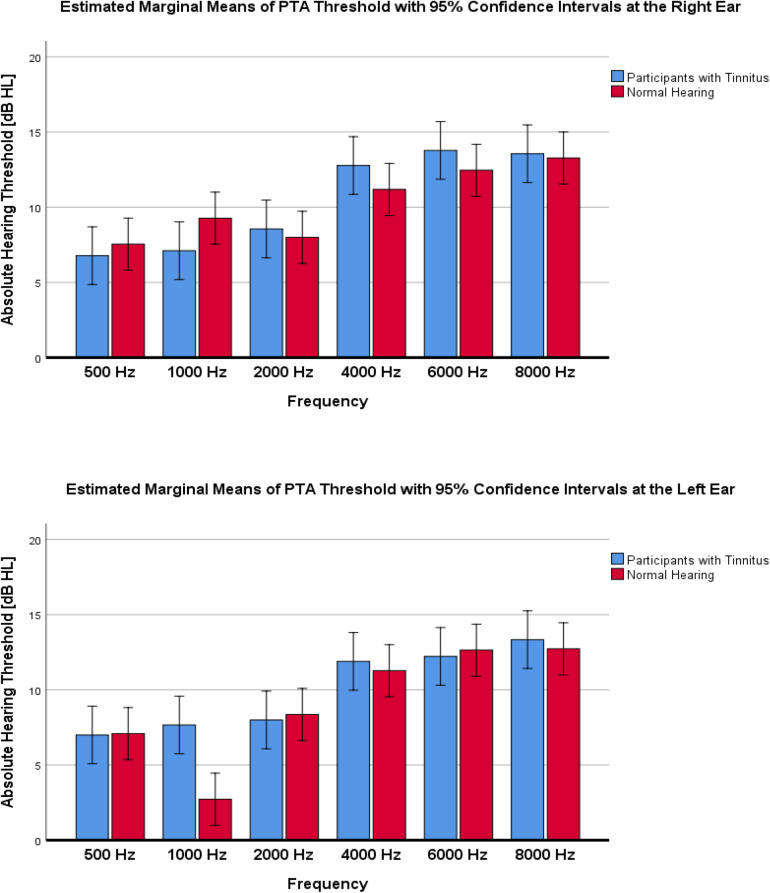
Mean of audiometric thresholds at 250–8000 Hz octave frequencies, recorded in decibels of hearing level (dB HL) in the left and right ear across the studied groups of participants with tinnitus vs. Normal Hearing. No significant differences between groups were found.

Pure tone audiometry measurements showed a normal to a very slight sloping sensorineural hearing loss above 4 kHz in both groups. Table 1 shows a composite audiogram of tinnitus and NH individuals who participated in the current study. However, the comparison of PTA values showed small and non-significant differences between the tinnitus (Mean ± 95% CI: 10.2 ± 0.8 dB HL) and normal control (Mean ± 95% CI: 9.7 ± 0.7 dB HL) groups (*F*_1_,_216_ = 1.818; *p* = 0.179; $\eta_{p}^{2}$ = 0.008; 95% CI [−0.52, 1.54]). Figure 3 indicates the mean of audiometric thresholds at 250–8000 Hz octave frequencies and depicts behavioral PTA threshold levels were ≤20 dB HL in octave frequencies of 250–2000 Hz and ≤25 dB HL in the 4000–8000 Hz frequency range.

**TABLE 1:** Composite audiogram of participants with tinnitus and NH individuals.

**Participants with tinnitus**								
**Frequency**		**0.5 kHz**	**1 kHz**	**2 kHz**	**4 kHz**	**6 kHz**	**8 kHz**	***P*-value**
Right ear	Mean ± 95% CI	6.78 ± 1.30	7.11 ± 1.51	8.56 ± 1.46	12.78 ± 1.52	13.78 ± 1.92	13.56 ± 2.42	0.82
Left ear	Mean ± 95% CI	7.00 ± 1.26	7.67 ± 1.53	8.00 ± 2.28	11.89 ± 1.78	12.22 ± 1.72	13.33 ± 3.99	
**Normal Hearing Group**								
**Frequency**		**0.5 kHz**	**1 kHz**	**2 kHz**	**4 kHz**	**6 kHz**	**8 kHz**	***P*-value**

Right ear	Mean ± 95% CI	7.55 ± 1.50	9.27 ± 1.81	8.00 ± 1.47	11.18 ± 0.94	12.45 ± 1.47	13.27 ± 1.49	0.54
Left ear	Mean ± 95% CI	7.09 ± 1.22	2.73 ± 1.54	8.36 ± 2.08	11.27 ± 1.77	12.64 ± 1.73	12.73 ± 2.03	

The mean duration of tinnitus was 41 months (95% CI = 18) in the tinnitus group ranging from 4 months to 15 years. The results of LMT, PMT, and MML for the group with tinnitus are (Mean ± 95% CI) 8.1 ± 2.6 dB SL, 6600 ± 1600 Hz, and 43.5 ± 13.0 dB HL, respectively.

**FIGURE 4:**
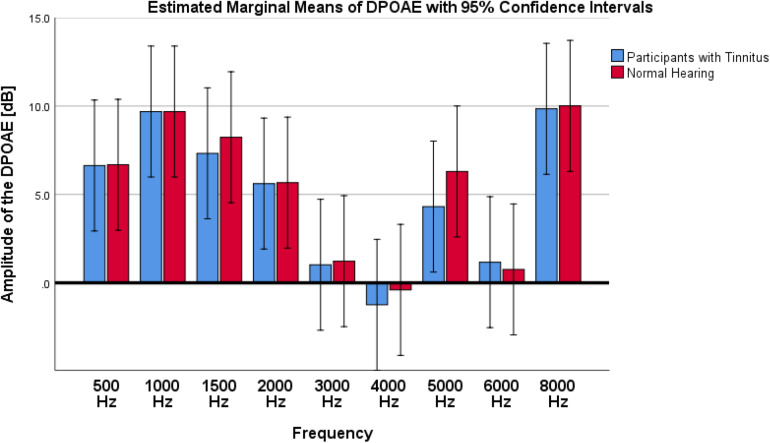
Results of DPOAE amplitude on participants with tinnitus and Normal Hearing group. As depicted in the graph, no significant differences were observed between tinnitus and NH control groups.

A comparison of the mean amplitudes of DPOAE showed a non-significant and small difference between two studied groups of tinnitus and NH control (*F*_1_,_144_ = 0.228; *p* = 0.633; $\eta_{p}^{2}$ = 0.002; 95% CI [−2.45, 1.6]). Figure 4 shows the results of DPOAE responses of tinnitus and NH groups.

### MMN Results

The effect of age was neither significant for the frontal subcomponent (*F*_1_,_36_ = 32.125; *p* = 0.691; $\eta_{p}^{2}$ = 0.004) and nor for the supratemporal subcomponent (*F*_1_,_36_ = 0.598; *p* = 0.444; $\eta_{p}^{2}$ = 0.016). For the frontal subcomponent, all the main effects except Frontality were significant (for Group: *F*_1_,_37_ = 36.746; *p* < 0.001; $\eta_{p}^{2}$ = 0.498; 95% CI [3.01, 3.5], for Laterality: *F*_2_,_74_ = 8.582; *p* < 0.001; ε = 0.932; $\eta_{p}^{2}$ = 0.188; 95% CI (3,z) [0.1, 0.7]; 95% CI (3,4) [−0.07, 0.53]; 95% CI (z,4) [−0.49, 0.15], for Deviant Central Frequency: *F_1_,_37_* = 19.094; *p* < 0.001; $\eta_{p}^{2}$ = 0.340; 95% CI [1.26, 1.75], and for Deviant Type: *F_7_,_37_* = 2.260; ε = 0.749; *p* < 0.05; $\eta_{p}^{2}$ = 0.066; 95% CI (Higher Frequency, Lower Frequency) [−0.25, 0.77]; 95% CI (Higher Frequency, Higher Intensity) [−0.75, 0.13]; 95% CI (Higher Frequency, Lower Intensity) [−1.1, −0.1]; 95% CI (Higher Frequency, Duration) [−1.87, −0.91]; 95% CI (Higher Frequency, Location (Left)) [−0.7, 0.24]; 95% CI (Higher Frequency, Location (Right)) [−0.79, 0.15]; 95% CI (Higher Frequency, Gap) [−1.46, −0.52]; 95% CI (Lower Frequency, Higher Intensity) [−1.06, −0.08]; 95% CI (Lower Frequency, Lower Intensity) [−1.4, −0.31]; 95% CI (Lower Frequency, Duration) [−2.17, −1.12]; 95% CI (Lower Frequency, Location (Left)) [−1, 0.03]; 95% CI (Lower Frequency, Location (Right)) [−1.1, −0.06]; 95% CI (Lower Frequency, Gap) [−1.77, −0.73]; 95% CI (Higher Intensity, Lower Intensity) [−0.77, 0.2]; 95% CI (Higher Intensity, Duration) [−1.54, −0.61]; 95% CI (Higher Intensity, Location (Left)) [−0.37, 0.54]; 95% CI (Higher Intensity, Location (Right)) [−0.46, 0.45]; 95% CI (Higher Intensity, Gap) [−1.13, −0.22]; 95% CI (Lower Intensity, Duration) [−1.31, −0.27]; 95% CI (Lower Intensity, Location (Left)) [−0.14, 0.88]; 95% CI (Lower Intensity, Location (Right)) [−0.23, 0.79]; 95% CI (Lower Intensity, Gap) [−0.9, 0.12]; 95% CI (Duration, Location (Left)) [0.67, 1.65]; 95% CI (Duration, Location (Right)) [0.57, 1.56]; 95% CI (Duration, Gap) [−0.1, 0.89]; 95% CI (Location (Left), Location (Right)) [−0.58, 0.39]; 95% CI (Location (Left), Gap) [−1.25, −0.28]; 95% CI (Location (Right), Gap) [−1.15, −0.19]). The interaction of the Frontality and Laterality was significant only between FC3 and FCz (*p* < 0.01) and other main effects or interactions were not significant.

For the supratemporal subcomponent, only Groups and Central Frequencies demonstrated significant main effect (for Group: *F*_1_,_37_ = 182.430; *p* < 0.001; $\eta_{p}^{2}$ = 0.409; 95% CI [−1.79, −1.37], and for Deviant Central Frequency: *F*_1_,_37_ = 28.176; *p* < 0.001; $\eta_{p}^{2}$ = 0.432; 95% CI [−0.98, −0.58]), and other main effects or interactions were not significant.

A permutation test with 10,000 permutations on temporal features of the MMN between groups and within groups showed that for stimuli and ROI channels altogether, the test rejected the null hypothesis (*p* < 0.001). Furthermore, permutation tests with 10,000 permutations on every stimulus, and every ROI channel between groups and within groups indicated that for all of the amplitudes with significant differences, the tests rejected the null hypothesis (*p* < 0.05).

#### Between Groups Comparisons

The ANOVA compared Peak Amplitude variable between two groups for the two central frequencies and different deviant types.

**FIGURE 5:**
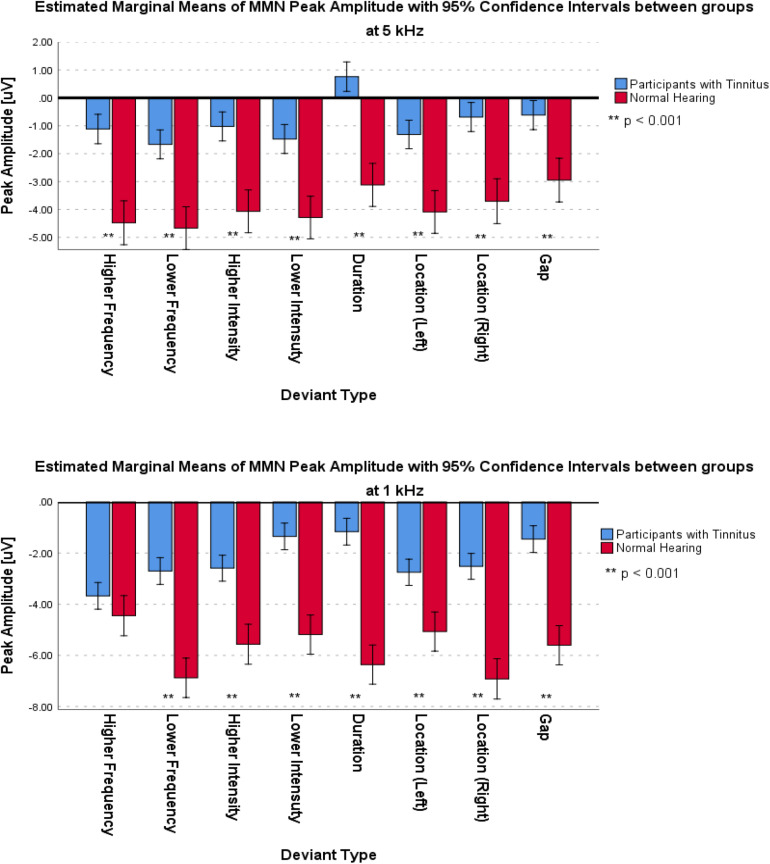
Estimated Marginal Means of MMN Peak Amplitude for frontal subcomponent with 95% Confidence Interval between groups at two central frequencies.

**FIGURE 6:**
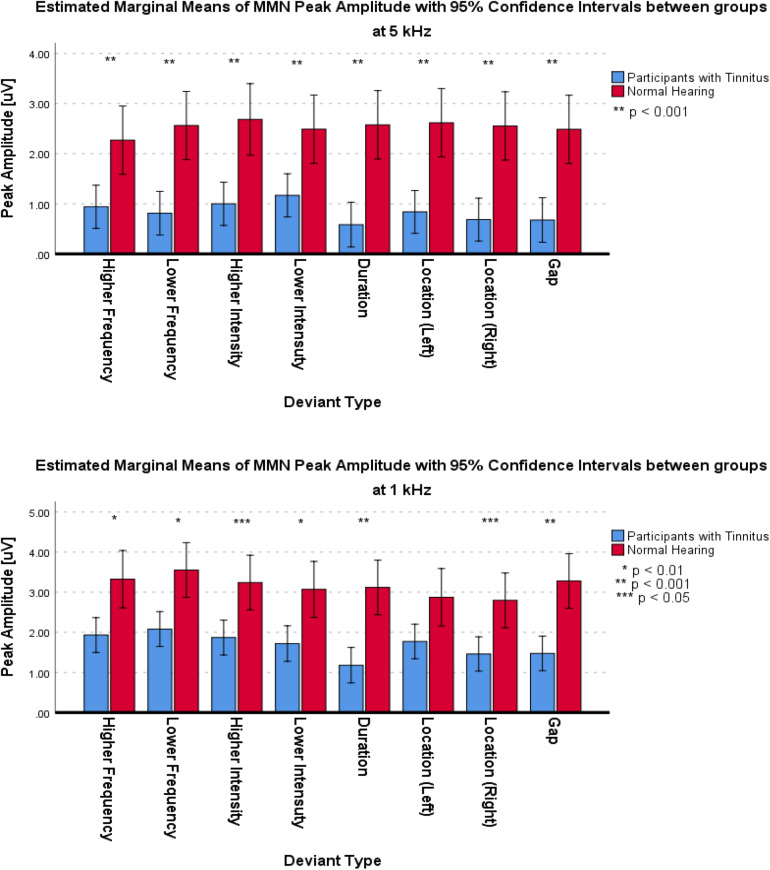
Estimated Marginal Means of MMN Peak Amplitude for supratemporal subcomponent with 95% Confidence Interval between groups at two central frequencies.

For the frontal subcomponent, the difference of all of the deviant types in two central frequencies except for the Higher Frequency deviant in 1 kHz central frequency were large and significantly less negative in the group with tinnitus (for Higher Frequency at 5 kHz: *F*_1_,_201_ = 78.442; *p* < 0.001; $\eta_{p}^{2}$ = 0.281; 95% CI [2.61, 4.11], for Lower Frequency at 5 kHz: *F*_1_,_210_ = 104.136; *p* < 0.001; $\eta_{p}^{2}$ = 0.332; 95% CI [2.43, 3.6], for Higher Intensity at 5 kHz: *F*_1_,_208_ = 113.557; *p* < 0.001; $\eta_{p}^{2}$ = 0.353; 95% CI [2.49, 3.63], for Lower Intensity at 5 kHz: *F*_1_,_209_ = 69.516; *p* < 0.001; $\eta_{p}^{2}$ = 0.250; 95% CI [2.14, 3.48], for Duration at 5 kHz: *F*_1_,_203_ = 172.700; *p* < 0.001; $\eta_{p}^{2}$ = 0.460; 95% CI [3.29, 4.46], for Location (Left) at 5 kHz: *F*_1_,_212_ = 92.174; *p* < 0.001; $\eta_{p}^{2}$ = 0.303; 95% CI [2.21, 3.36], for Location (Right) at 5 kHz: *F*_1_,_201_ = 106.887; *p* < 0.001; $\eta_{p}^{2}$ = 0.347; 95% CI [2.44, 3.6], for Gap at 5 kHz: *F*_1_,_204_ = 58.714; *p* < 0.001; $\eta_{p}^{2}$ = 0.223; 95% CI [1.73, 2.94], for Lower Frequency at 1 kHz: *F*_1_,_205_ = 35.083; *p* < 0.001; $\eta_{p}^{2}$ = 0.146; 95% CI [2.77, 5.56], for Higher Intensity at 1 kHz: *F*_1_,_212_ = 36.383; *p* < 0.001; $\eta_{p}^{2}$ = 0.146; 95% CI [2, 3.95], for Lower Intensity at 1 kHz: *F*_1_,_208_ = 32.708; *p* < 0.001; $\eta_{p}^{2}$ = 0.136; 95% CI [2.54, 5.22], for Duration at 1 kHz: *F*_1_,_206_ = 113.681; *p* < 0.001; $\eta_{p}^{2}$ = 0.356; 95% CI [4.23, 6.16], for Location (Left) at 1 kHz: *F*_1_,_212_ = 13.867; *p* < 0.001; $\eta_{p}^{2}$ = 0.061; 95% CI [1.09, 3.56], for Location (Right) at 1 kHz: *F*_1_,_212_ = 67.292; *p* < 0.001; $\eta_{p}^{2}$ = 0.241; 95% CI [3.32, 5.43], and for Gap at 1 kHz: *F*_1_,_206_ = 53.450; *p* < 0.001; $\eta_{p}^{2}$ = 0.206; 95% CI [3.04, 5.3]). Figure 5 shows the estimated Marginal Means of the MMN Peak Amplitude for the frontal subcomponent with 95% Confidence Intervals between groups at two central frequencies. For the supratemporal subcomponent, all of the deviant types with 5 kHz central frequencies (for Higher Frequency at 5 kHz: *F*_1_,_75_ = 19.526; *p* < 0.001; $\eta_{p}^{2}$ = 0.207; 95% CI [−1.93, −0.72], for Lower Frequency at 5 kHz: *F*_1_,_74_ = 28.228; *p* < 0.001; $\eta_{p}^{2}$ = 0.276; 95% CI [−2.41, −1.09], for Higher Intensity at 5 kHz: *F*_1_,_73_ = 29.268; *p* < 0.001; $\eta_{p}^{2}$ = 0.286; 95% CI [−2.31, −1.06], for Lower Intensity at 5 kHz: *F*_1_,_75_ = 20.280; *p* < 0.001; $\eta_{p}^{2}$ = 0.213; 95% CI [−1.91, −0.73], for Duration at 5 kHz: *F*_1_,_71_ = 43.748; *p* < 0.001; $\eta_{p}^{2}$ = 0.381; 95% CI [−2.59, −1.38], for Location (Left) at 5 kHz: *F*_1_,_76_ = 30.032; *p* < 0.001; $\eta_{p}^{2}$ = 0.283; 95% CI [−2.43, −1.13], for Location (Right) at 5 kHz: *F*_1_,_76_ = 28.268; *p* < 0.001; $\eta_{p}^{2}$ = 0.271; 95% CI [−2.57, −1.16], and for Gap at 5 kHz: *F*_1_,_72_ = 51.064; *p* < 0.001; $\eta_{p}^{2}$ = 0.415; 95% CI [−2.32, −1.3]) and all of the deviant types with 1 kHz central frequencies, except for the Location (Left) deviant (for Higher Frequency at 1 kHz: *F*_1_,_72_ = 11,625; *p* < 0.002; $\eta_{p}^{2}$ = 0.139; 95% CI [−2.21, −0.57], for Lower Frequency at 1 kHz: *F*_1_,_74_ = 7.037; *p* < 0.01; $\eta_{p}^{2}$ = 0.087; 95% CI [−2.58, −0.36], for Higher Intensity at 1 kHz: *F_1_,_74_* = 8.796; *p* < 0.01; $\eta_{p}^{2}$ = 0.106; 95% CI [−2.3, −0.45], for Lower Intensity at 1 kHz: *F*_1_,_71_ = 10.975; *p* < 0.001; $\eta_{p}^{2}$ = 0.134; 95% CI [−2.14, −0.53], for Duration at 1 kHz: *F*_1_,_72_ = 23.410; *p* < 0.001; $\eta_{p}^{2}$ = 0.245; 95% CI [−2.74, −1.14], for Location (Left) at 1 kHz: *F*_1_,_73_ = 2.599; *p* > 0.111; $\eta_{p}^{2}$ = 0.034; 95% CI [−2.47, 0.27], for Location (Right) at 1 kHz: *F*_1_,_76_ = 6.267; *p* < 0.015; $\eta_{p}^{2}$ = 0.076; 95% CI [−2.41, −0.27], and for Gap at 1 kHz: *F*_1_,_75_ = 19.002; *p* < 0.001; $\eta_{p}^{2}$ = 0.202; 95% CI [−2.64, −0.98]) had large differences and the mean MMN peak amplitudes were significantly lower in the group with tinnitus. Figure 6 shows the estimated Marginal Means of the MMN Peak Amplitude for the supratemporal subcomponent with 95% Confidence Intervals between groups at two central frequencies.

**FIGURE 7:**
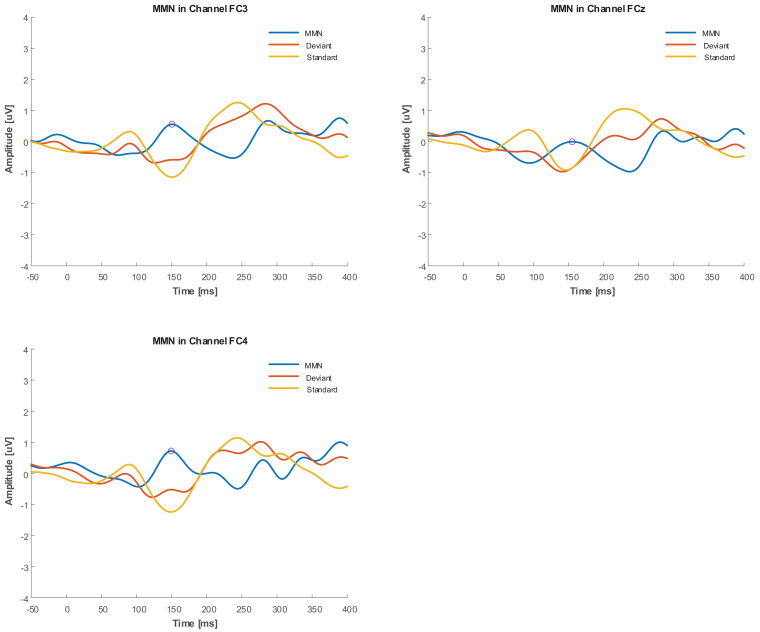
Grand average of duration deviant in FC3, FCz, and FC4 channels for Group with tinnitus. PMMR is indicated by circles.

As an *a posteriori* observation, the duration deviant with 5 kHz central frequency in the group with tinnitus elicited an MMN that had a positive mean value with negative second derivative (concave down), similar to the Positive Mismatch Response (PMMR) (Näätänen et al., 2014). The grand averages in Figure 7 show this effect for the duration deviant at FC3, FCz, and FC4.

#### Within Group Comparisons

The ANOVA used the Peak Amplitude variable to implement the comparison within the two groups in the two central frequencies and different deviant types.

**FIGURE 8:**
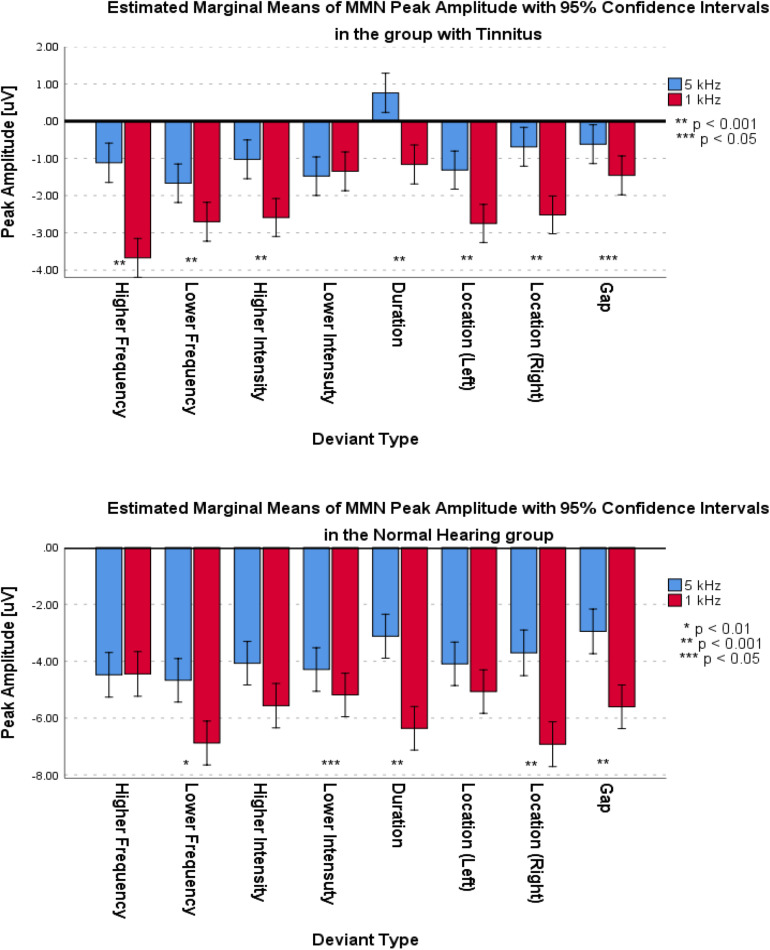
Estimated Marginal Means of MMN Peak Amplitude for frontal subcomponent with 95% Confidence Interval within groups.

**FIGURE 9:**
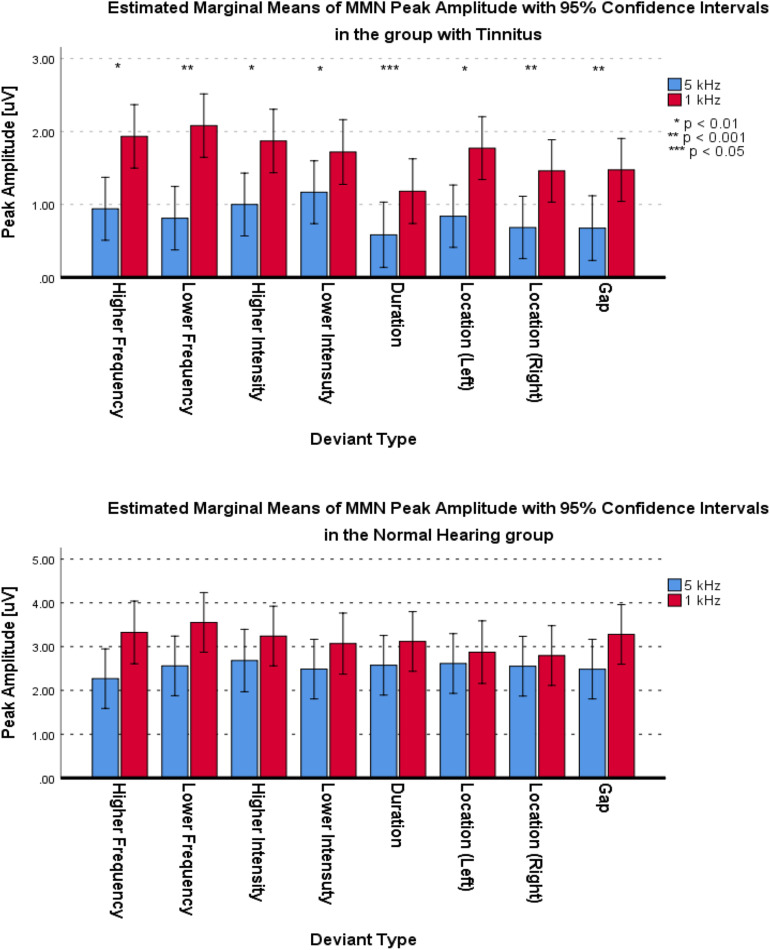
Estimated Marginal Means of MMN Peak Amplitude for supratemporal subcomponent with 95% Confidence Interval within groups.

For the frontal subcomponent, all of the deviant types except for the Lower Intensity deviant in the group with tinnitus had medium differences and significantly less negative peak amplitudes at 5 kHz central frequency (for Higher Frequency: *F*_1_,_281_ = 40.527; *p* < 0.001; $\eta_{p}^{2}$ = 0.126; 95% CI [1.75, 3.33], for Lower Frequency: *F*_1_,_286_ = 6.614; *p* < 0.012; $\eta_{p}^{2}$ = 0.023; 95% CI [0.24, 1.85], for Higher Intensity: *F*_1_,_292_ = 113.557; *p* < 0.001; $\eta_{p}^{2}$ = 0.091; 95% CI [0.99, 2.14], for Lower Intensity: *F*_1_,_287_ = 0.133; *p* > 0.716; $\eta_{p}^{2}$ = 0.000; 95% CI [−1.1, 0.75], for Duration: *F*_1_,_280_ = 33.479; *p* < 0.001; $\eta_{p}^{2}$ = 0.107; 95% CI [1.26, 2.58], for Location (Left): *F*_1_,_294_ = 14.065; *p* < 0.001; $\eta_{p}^{2}$ = 0.046; 95% CI [0.68, 2.19], for Location (Right): *F*_1_,_292_ = 31.541; *p* < 0.001; $\eta_{p}^{2}$ = 0.097; 95% CI [1.18, 2.48], and for Gap: *F*_1_,_283_ = 4.683; *p* < 0.05; $\eta_{p}^{2}$ = 0.016; 95% CI [0.07, 1.55]). In the NH group, all of the deviant types except for the Higher Frequency, Higher Intensity, and Location (Left) deviants were significantly less negative with small differences at 5 kHz central frequency (for Higher Frequency: *F*_1_,_124_ = 0.015; *p* > 0.309; $\eta_{p}^{2}$ = 0.000; 95% CI [−0.72, 0.64], for Lower Frequency: *F*_1_,_129_ = 10.567; *p* < 0.002; $\eta_{p}^{2}$ = 0.076; 95% CI [0.86, 3.54], for Higher Intensity: *F*_1_,_128_ = 7.122; *p* > 0.05; $\eta_{p}^{2}$ = 0.053; 95% CI [0.38, 2.59], for Lower Intensity: *F*_1_,_130_ = 3.940; *p* < 0.05; $\eta_{p}^{2}$ = 0.029; 95% CI [0, 1.79], for Duration: *F*_1_,_129_ = 54.929; *p* < 0.001; $\eta_{p}^{2}$ = 0.299; 95% CI [2.37, 4.11], for Location (Left): *F*_1_,_130_ = 2.900; *p* > 0.090; $\eta_{p}^{2}$ = 0.022; 95% CI [−0.16, 2.11], for Location (Right): *F*_1_,_121_ = 33.488; *p* < 0.001; $\eta_{p}^{2}$ = 0.217; 95% CI [2.09, 4.28], and for Gap: *F*_1_,_127_ = 28.514; *p* < 0.05; $\eta_{p}^{2}$ = 0.183; 95% CI [1.66, 3.64]). Figure 8 shows the estimated Marginal Means of MMN Peak Amplitude for the frontal subcomponent with 95% Confidence Interval within groups. For the supratemporal subcomponent, all of the deviant types in the group with tinnitus were significantly lower with medium differences at 5 kHz central frequency (for Higher Frequency: *F*_1_,_107_ = 17.996; *p* < 0.001; $\eta_{p}^{2}$ = 0.144; 95% CI [−1.46, −0.52], for Lower Frequency: *F*_1_,_106_ = 13.726; *p* < 0.001; $\eta_{p}^{2}$ = 0.115; 95% CI [−1.95, −0.59], for Higher Intensity: *F*_1_,_107_ = 11.211; *p* < 0.002; $\eta_{p}^{2}$ = 0.095; 95% CI [−1.39, −0.35], for Lower Intensity: *F*_1_,_105_ = 5.879; *p* < 0.01; $\eta_{p}^{2}$ = 0.059; 95% CI [−1.01, −0.1], for Duration: *F*_1_,_101_ = 4.830; *p* < 0.05; $\eta_{p}^{2}$ = 0.046; 95% CI [−1.13, −0.05], for Location (Left): *F*_1_,_109_ = 6.122; *p* < 0.01; $\eta_{p}^{2}$ = 0.053; 95% CI [−1.69, −0.18], for Location (Right): *F*_1_,_110_ = 6.936; *p* < 0.01; $\eta_{p}^{2}$ = 0.059; 95% CI [−1.36, −0.19], and for Gap: *F*_1_,_105_ = 11.057; *p* < 0.01; $\eta_{p}^{2}$ = 0.095; 95% CI [−1.28, −0.32]). ANOVA revealed no significant difference between central frequencies in the NH group for the supratemporal subcomponent. Figure 9 shows the estimated Marginal Means of MMN Peak Amplitude for the supratemporal subcomponent with 95% Confidence Intervals within groups.

## Discussion

This study targeted the comparison of the neural correlates of the pre-attentive central auditory processing related to specific frequencies of acoustic stimuli (as indexed by the MMN responses) in the participants with tinnitus and NH controls. A multi-feature paradigm with two central frequencies extracted MMN in tinnitus and NH groups. The results show that the frontal subcomponent of MMN exhibited a large difference with lower amplitude of peak negativity in the group of persons with tinnitus compared to the group of persons with NH. For the supratemporal subcomponent, the results indicate a large difference with significantly lower MMN peak amplitude in the group with tinnitus. These results are in agreement with two previous studies that reported lower MMN amplitude in the group with tinnitus compared to the NH group (Mahmoudian et al., 2013; Yang et al., 2013). One of these studies used only frequency deviants (Yang et al., 2013), whereas the other utilized the difference between groups in frequency, duration, and silent gap deviants (Mahmoudian et al., 2013). However, previous studies that utilized MMN in participants with tinnitus did not investigate two different central frequencies and did not evaluate the effect of tinnitus on MMN subcomponents. The current study shows a deficit in Pre-attentive Mismatch Response and Involuntary Attention (subconsciousness) according to less negative peak amplitude of frontal subcomponent in the group with tinnitus. Also, the lower peak amplitude of the supratemporal subcomponent may indicate a deficit in the change detection aspect in the group with tinnitus. Another interesting result is the higher difference between the mean peak amplitude in the supratemporal subcomponent at the central frequency of 5 kHz in the between groups. This difference may be due to increased MMN change detection deficits in the higher frequencies closer to the tinnitus perceived frequency.

Despite the statistically large and significant difference in the age of the subjects in the group with tinnitus compared to the NH group, based on various studies, this difference could not have caused a significant change in cortical auditory responses. Salvi et al. (2018) reported that age-related hearing loss can affect roughly 35% of people over the age of 70 years old. Another study revealed a loss of 30–40% of the Outer Hair Cell (OHC) population throughout the audiometric frequency range (0.25–8.0 kHz) in individuals over 60 years old (Wu et al., 2019). In the current study, the maximum age of any subject in either group was less than 50 years; thus, the age-related hidden hearing loss does not seem to significantly affect the MMN. Additionally, according to previous studies, it is difficult to determine a quantitative indicator of the age-related changes of cochlear hair cell survival in humans. In fact, many factors may underlie the discordant studies relating noise to hidden hearing loss, including inaccuracies in self-reporting of lifetime noise exposure, potential confounding effects of age and central auditory system compensation mechanisms, and the effects of different underlying mechanisms on physiological and behavioral measures of the hearing (Salvi et al., 2018; Wu et al., 2019). Surely, an appropriate conclusion about the effect of age-related hearing loss in hearing pathways will require careful selection of comprehensive age groups with detailed evaluations.

A previous study reported the role of hidden hearing loss using high-frequency PTA and ECochG results between two groups of normal-hearing participants (PTA 125 Hz to 8 kHz) with and without tinnitus (Kara et al., 2020). Kujawa and Liberman (2015) considered cochlea synaptopathy as an origin for the hidden hearing loss in tinnitus. According to another study (Schaette and McAlpine, 2011), deafferentation of a substantial fraction of the auditory nerve fibers, as observed in the mice, following “temporary” hearing loss due to exposure to loud noise (Kujawa and Liberman, 2009), could trigger the development of a neural correlate of tinnitus in the central auditory structures. To limit the impact of any hidden hearing loss (cochlear synaptopathy) on the studied groups, the current study excluded the participants with exposure to loud noise, history of taking ototoxic medication, low speech discrimination score, presbycusis, and absence of DPOAE responses. However, despite the careful controls and the fact that the amount of impact was quite small, the current investigation could not entirely exclude the role of any hidden hearing loss and the subcortical origins of tinnitus on the MMN responses and age-related subcortical changes.

Some of the previous studies reported that the ability to discriminate speech in background noise declines with aging even when there is no significant increase in audiometric thresholds (Dubno et al., 1984; Rajan and Cainer, 2008). Furthermore, animal studies revealed that the aging process, in the absence of significant noise exposure, is associated with the loss of auditory nerve fibers (Plack et al., 2014). Evidence from psychophysical and electrophysiological studies suggests that the coding of the temporal aspects of sounds declines with aging (He et al., 2008; Hopkins and Moore, 2011; Moore et al., 2012; Clinard and Tremblay, 2013; Marmel et al., 2013; King et al., 2014). Moreover, the results of animal experiments indicate that noise exposure may cause substantial cochlear neuropathy without affecting the audiogram (Plack et al., 2014). Other pieces of evidence from human studies revealed that exposure to noise might be associated with deficits in suprathreshold auditory discrimination and neural temporal coding, in the absence of a reduction in hearing sensitivity (Schaette and McAlpine, 2011; Plack et al., 2014). Based on the findings of the above-mentioned studies, the presence of cochlear synaptopathy or hidden hearing loss in the studied tinnitus group can lead to a deficit in neural coding of both temporal fine structure and temporal envelope in humans. As the MMN responses evaluate the neural correlates of auditory discrimination and automated sensory memory, analyzing the MMN component may elucidate the cerebral processes in tinnitus subjects that occur during auditory perception and cognition (Mahmoudian et al., 2013). Results from the present study reveal that individuals with tinnitus may have defective auditory discrimination and sensory memory in the central auditory pathways. The existence of these findings may also suggest that hidden hearing loss in central auditory pathways may alter auditory sensory memory and suprathreshold discrimination. In fact, these perceptual consequences, in particular the coding process of the temporal aspects of sounds, may, in turn, create a deficit in the MMN responses among persons with tinnitus. The current study did not measure the suprathreshold auditory discrimination in the presence of background noise. Hence, the present investigation can neither prove nor disprove the role of cochlear synaptopathy in tinnitus participants concerning the decline of the MMN results.

This study conducted novel within-group comparisons, for which the mean of MMN peak amplitude in higher frequencies demonstrated a decrease in both groups. In contrast, higher number of deviants in the group with tinnitus were less negative at 5 kHz central frequency for the frontal subcomponent with medium differences compared with 1 kHz central frequency. In fact, the presence of more deviants with less negative peak amplitudes may indicate the decrease of Pre-attentive Mismatch Response and Involuntary Attention (subconsciousness) in higher frequencies. Nevertheless, an important observation is in the supratemporal subcomponent within groups. The results showed no meaningful difference between the MMN peak amplitude at two central frequencies in the NH group while there was a medium difference and significant decrease in the group with tinnitus at 5 kHz compared to 1 kHz central frequencies. On the other hand, the lack of significant differences in the NH group may demonstrate that change detection in the NH group is independent of the frequency of the stimuli. In contrast, the significant decrease of the MMN peak amplitude in the group with tinnitus may indicate that increasing the frequency of the stimuli decreases the change detection ability. In view of the novel analytical results presented in the current study, it is important to note that previous studies have not reported any comparison of MMN subcomponents in different central frequencies within the NH group and the group with tinnitus.

The sensory memory hypothesis may explain another interesting observation, namely, with regard to the difference between and within the groups. According to the sensory memory hypothesis and the results of the current study, the change detection process, which primarily takes place in the auditory cortex and frontal cortex (Näätänen et al., 2005), and pre-attentive memory may have deficits in the group with tinnitus and may cause lower MMN response. Some of the previous studies on the MMN response in participants with tinnitus have used this interpretation to explain the difference between tinnitus and NH groups (Mahmoudian et al., 2013; Yang et al., 2013; Li et al., 2016). As such, within-group comparisons in the supratemporal subcomponent, indicating the change detection, show a lower function of pre-attentive memory in the deviants with higher central frequencies. Importantly, the pre-attentive function is attenuated in the group with tinnitus while NH group shows no significant attenuation. The hypothesis is further related to the predictive coding model, which assumes that the brain forms a predictive model of the perceived inputs. Hence, when the brain receives a non-regular input, a prediction error happens that leads to the MMN response (Friston, 2005; Fishman, 2014). A study by Sedley et al. (2016) used the predictive coding model and suggested that tinnitus occurs when sensory prediction error of spontaneous neural activity in the auditory pathway rises sufficiently to override the perception of silence. A lower MMN response may mean lower prediction error for the deviant stimuli, and the reduction of prediction error for deviant stimuli may indicate lower prediction error for silence, which in turn may cause tinnitus. Also, according to within-groups comparisons in the supratemporal subcomponent, prediction error in higher central frequencies is lower in the group with tinnitus. The reduction of the prediction error may decrease the difference between the predicted backward input and the actual input, including the difference between the inputs of silence. Therefore, the prediction error of spontaneous neural activity may be high enough in higher frequencies to exceed the error of silence. Hence, higher prediction error of spontaneous neural activity as compared to the prediction error of silence may be considered as the cause of tinnitus occurrence in higher frequencies.

The current study utilized the sensory-memory hypothesis, predictive error modeling, and memory network to interpret the generation of tinnitus. The index of this interpretation was the MMN. In accordance with the present investigation, a recent study found aberrant functional connectivity in the auditory and non-auditory cortex, especially in the superior temporal gyrus (Cai et al., 2020). Other studies suggested different interpretations of the cause of tinnitus occurrence. One of these studies suggested that having a lesion of the auditory periphery may be a trigger for the tinnitus, and lack of an inhibitory feedback loop in paralimbic regions may cause the tinnitus signal to relay to the auditory cortex (Rauschecker et al., 2010). Another study proposed that a feedback pathway from the limbic system to the auditory system at the subcortical level may suppress the tinnitus signal (Kraus and Canlon, 2012). A more recent study used functional magnetic resonance imaging (fMRI) to investigate the connection between the auditory network and the limbic system (Cai et al., 2019). Husain et al. (2011) suggested a relation between the differential engagement of auditory attention and short-term memory network to the neural bases of chronic tinnitus with hearing loss compared to hearing loss alone. However, the study did not investigate the tinnitus generation in persons without hearing loss. Reduced neural output from the cochlea due to a hidden hearing loss may represent another indicator of the tinnitus initiation, causing a homeostatic response of neurons in the auditory system and a perception of the tinnitus (Schaette and McAlpine, 2011). These studies suggest a role for memory, emotion, distress, and other networks separately for the occurrence of tinnitus. Integrating these networks and the resulting neural interactions may lead to a better understanding of the origin of tinnitus as well as better strategies for neural rehabilitation.

The current study implemented a multi-feature paradigm to investigate the effect of tinnitus on MMN subcomponents using the stimuli with two different central frequencies in the group with tinnitus compared to the NH group. The central frequency of 5 kHz was closer to perceived tinnitus frequencies. The present investigation evaluated the effects of five independent variables, namely, Group, Central Frequencies, Deviant types, Frontality, and Laterality, on the MMN peak amplitudes. A mixed ANOVA with a critical α of 0.05 calculated the main effects and interactions. The statistical results showed a significantly less negative MMN peak amplitude with large differences in the group with tinnitus compared to the NH group at the central frequency of 5 kHz for the frontal MMN subcomponent. Also, the results illustrated a meaningful lower MMN peak amplitude with large differences in the central frequency of 5 kHz for the supratemporal MMN subcomponent in the group with tinnitus. The results are in accordance with the previous investigations, although those studies did not investigate the MMN for different subcomponents. The novel part of the current study is the within-group comparisons of the central frequencies. The results indicated that there are meaningful, less negative MMN peaks at the central frequency of 5 kHz with small to medium differences for both groups in the frontal subcomponent. Moreover, the group with tinnitus revealed significantly lower peak amplitude with medium differences at 5 kHz compared to 1 kHz for the supratemporal MMN subcomponent while the NH group did not register any significant difference. Lastly, based on the predictive coding model, results of the within-groups in the higher frequencies suggested that for persons with tinnitus the difference between the amplitude of inputs from the lower levels of the brain and the predicted input from the higher levels is reduced. In turn, as the prediction error of silence decrease, the prediction error of spontaneous neural activity in the auditory pathway may exceed the silent prediction error, and as a result, the possibility of occurrence of the tinnitus may increase.

## Data Availability Statement

The datasets generated for this study are available on request to the corresponding author.

## Ethics Statement

The studies involving human participants were reviewed and approved by the Ethics Committee of Iran University of Medical Sciences (IUMS) through Ethics Code of IR.IUMS.REC.1393.9011369004. Furthermore, participants provided written informed consent. The patients/participants provided their written informed consent to participate in this study.

## Author Contributions

AA contributed to design of the work, data acquisition, analysis of data, and draft of the manuscript. MJ contributed to the concept and design of the work, supervising the analysis, and revising and finalizing the manuscript. SM contributed to the concept and design of the work, supervision of experiments, and revising the manuscript. All authors contributed to the article and approved the submitted version.

## Conflict of Interest

The authors declare that the research was conducted in the absence of any commercial or financial relationships that could be construed as a potential conflict of interest.

## Footnotes

1 http://www.path-medical.de
